# Complex sputum microbial composition in patients with pulmonary tuberculosis

**DOI:** 10.1186/1471-2180-12-276

**Published:** 2012-11-23

**Authors:** Zelin Cui, Yuhua Zhou, Hong Li, Yan Zhang, Shulin Zhang, Shenjie Tang, Xiaokui Guo

**Affiliations:** 1 Department of Medical Microbiology and Parasitology, Shanghai Jiao Tong University School of Medicine, 200025, Shanghai, China; 2 Tuberculosis Centre for Diagnosis and Treatment, Shanghai Pulmonary Hospital, Tongji University School of Medicine, Shanghai 200433, China

**Keywords:** Pulmonary tuberculosis, Sputum, Microbiota, Diversity

## Abstract

**Background:**

An increasing number of studies have implicated the microbiome in certain diseases, especially chronic diseases. In this study, the bacterial communities in the sputum of pulmonary tuberculosis patients were explored. Total DNA was extracted from sputum samples from 31 pulmonary tuberculosis patients and respiratory secretions of 24 healthy participants. The 16S rRNA V3 hyper-variable regions were amplified using bar-coded primers and pyro-sequenced using Roche 454 FLX.

**Results:**

The results showed that the microbiota in the sputum of pulmonary tuberculosis patients were more diverse than those of healthy participants (*p*<0.05). The sequences were classified into 24 phyla, all of which were found in pulmonary tuberculosis patients and 17 of which were found in healthy participants. Furthermore, many foreign bacteria, such as *Stenotrophomonas*, *Cupriavidus*, *Pseudomonas*, *Thermus*, *Sphingomonas*, *Methylobacterium*, *Diaphorobacter*, *Comamonas*, and *Mobilicoccus,* were unique to pulmonary tuberculosis patients.

**Conclusions:**

This study concluded that the microbial composition of the respiratory tract of pulmonary tuberculosis patients is more complicated than that of healthy participants, and many foreign bacteria were found in the sputum of pulmonary tuberculosis patients. The roles of these foreign bacteria in the onset or development of pulmonary tuberculosis shoud be considered by clinicians.

## Background

Chronic pulmonary tuberculosis poses a global health emergency. It has been known for many centuries and is mainly caused by the bacillus *Mycobacterium tuberculosis*. Many reports have revealed co-infection with different strains or species of *Mycobacterium* in pulmonary tuberculosis patients. Mixed infection with Beijing and non-Beijing strains of *M.tuberculosis*[1] has been reported to mediate the increased reinfection rate in regions with a high incidence of tuberculosis. Similarly, MAC (*Mycobacterium avium complex*) and *M.tuberculosis* coexist in some patients with combined mycobacterial infections [2]. The systems biology concept of persistent infection is that infectious diseases reflect an equilibrium between the host and the pathogen that is established and maintained by a broad network of interactions. These interactions occur across scales that range from molecular to cellular, to whole organism and population levels [3].

The development of nucleotide sequencing has helped reveal the importance of microbiota to human health [4]. For example, community and microbial ecology-based pathogenic theories have been introduced to explain the relationship between dental plaque and the host [5]. The urine microbiomes of men with sexually transmitted infection were found to be dominated by fastidious, anaerobic and uncultivable bacteria [6]. Furthermore, the microbiota interact with nutrients and host biology to modulate the risk of obesity and associated disorders, including diabetes, obesity inflammation, liver diseases and bacterial vaginosis (BV) [7-10]. Patients with neonatal necrotising enterocolitis have lower microbiota diversity, which is asscociated with an increase in the abundance of *Gammaproteobacteria*[11]*.* Ichinohe *et al* revealed that microbiota can regulate the immune defence against respiratory tract influenza A virus infection [12]. Ehlers and Kaufmann also emphasised the association between chronic diseases and dysbiosis or a disturbed variability of the gut microbiome [13]. In light of the recent discovery of cystic fibrosis associated lung microbiota, Delhaes and Monchy *et al* discussed the microbial community as a unique pathogenic entity [14]. Huang and Lynch emphasised that microbiota, as a collective entity, may contribute to pathophysiologic processes associated with chronic airway disease [15]. Robinson *et al* also suggested the conservation or restoration of the normal community structure and function of host-associated microbiota should be included in the prevention and treatment of human disease [16]. In summary, microbiota are very important to human health, Understanding the microbial composition in the respiratory tract of pulmonary tuberculosis patients may enhance our awareness of microbiota as a collective entity or even collective pathogenic entity, and the role this entity plays in the onset and development of pulmonary tuberculosis.

In this work, we collected 31 sputum samples from pulmonary tuberculosis patients from Shanghai Pulmonary Hospital, and 24 respiratory secretion samples from healthy participants in Shanghai, China as controls, and investigated the composition of the microbiota in the lower respiratory tract of pulmonary tuberculosis patients.

## Results and discussion

### Results

#### Sequence diversity

The 454 pyro-sequencing method was used to analysis a total of 71928 PCR amplicons in the samples from pulmonary tuberculosis patients and healthy participants, The amplicons averaged approximately 200 bp in length, and the average number of sequences per sample was 1307.8. The mean Shannon diversity and evenness indices in the pulmonary tuberculosis samples were 6.1926 (SD, 0.8093) and 0.9615 (SD, 0.0177), respectively. Both indices were significantly higher than those in the healthy participants, which were 5.5145 (SD, 0.6545) (*p*=0.006) and 0.9341 (SD, 0.0216) (*p*=0.000) , respectively.

#### Clustering analysis of the respiratory tract microbiota can separate healthy participants from pulmonary tuberculosis patients

**Figure 1:**
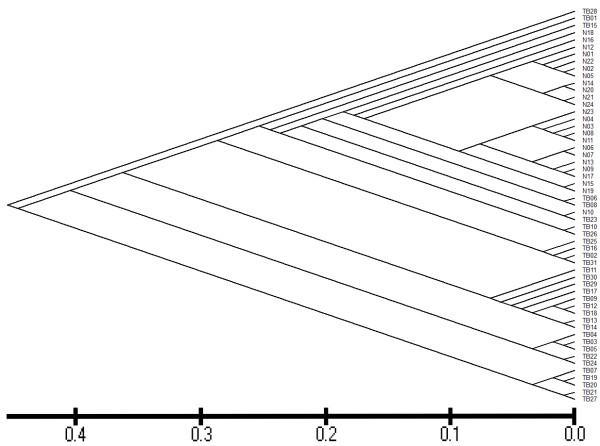
**Bacterial communities grouped by individual.** Each terminal branch represents the total bacterial community detected in one enrolled subject. All nodes were recovered at 100% using the Jackknife method. Names beginning with “N” represent samples from healthy participants, while those beginning with “TB” represent samples from patients with pulmonary tuberculosis.

The similarities between the respiratory tract secretion microbiota of the healthy participants and sputum microbiota of the pulmonary tuberculosis patients were estimated by calculating UniFrac distances. Figure 1 shows that the healthy participants were clustered together, while the pulmonary tuberculosis patients were divided into several different sub-branches.

**Figure 2:**
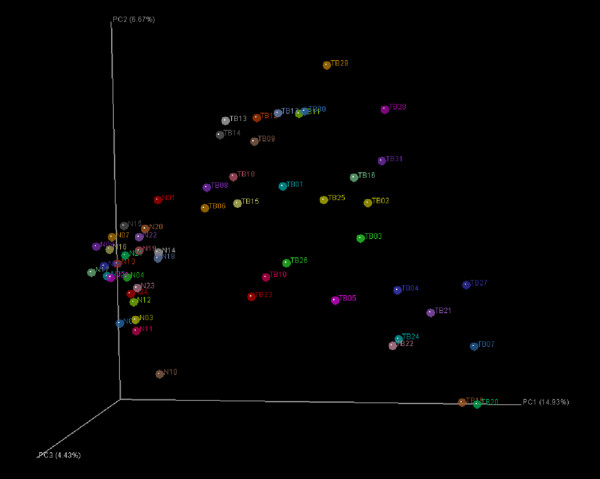
**UniFrac community comparison of healthy participants and patients with pulmonary tuberculosis.** The sputum microbiomes were clustered using un-weighted UniFrac. The percentage of variation explained by each principal component is indicated on the axis. The circles with names beginning with “N” represent samples from healthy participants, while those beginning with “TB” correspond to samples from patients with pulmonary tuberculosis.

**Figure 3:**
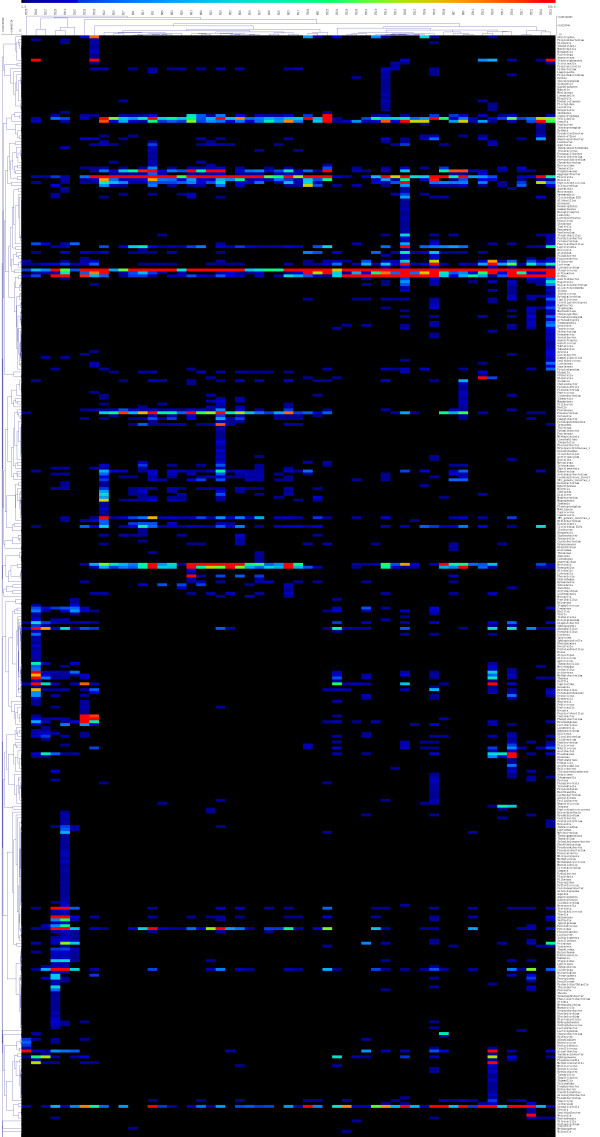
**Hierarchical clustering of sputum microbial composition at the genus level.** The names of some of the most abundant genera corresponding to terminal taxa depicted in the heatmap are listed to the right of the figure. Subjects listed at the top and right of the heatmap indicate microbiome and genus relationships, respectively. Names beginning with “N” represent samples from healthy participants, while those beginning with “TB” correspond to samples from patients with pulmonary tuberculosis.

As shown in Figure 2, clustering after principal coordinate analysis (PCoA) of the UniFrac distance demonstrated a strong clustering of healthy participants away from pulmonary tuberculosis patients. To better characterise the sputum microbiomes, the sequences were sorted to the genera level. A total 614 genera were observed; 235 genera were observed in healthy participants, and 564 genera were found in pulmonary tuberculosis patients, although more than half of these accounted for only a small fraction of the total sequences. As shown in Figure 3, *Streptococcus*, *Granulicatella*, *Actinomyces*, *Prevotella*, and *Veillonella* were predominant in the microbiota of both healthy participants and pulmonary tuberculosis patients. In contrast, *Anoxybacillus, Klebsiella, Acinetobacter*, *Pilibacter*, *Abiotrophia*, *Paucisalibacillus*, and *Rothia* were more abundant in pulmonary tuberculosis patients than healthy participants. *Neisseria*, *Porphyromonas*, *TM7_genera_incertae_sedis*, *Parvimonas*, *Campylobacter*, *Haemophilus*, and *Fusobacterium* were less common in pulmonary tuberculosis patients than healthy participants. Furthermore, *Stenotrophomonas*, *Cupriavidus*, *Pseudomonas*, *Thermus*, *Sphingomonas*, *Brevundimonas*, *Brevibacillus*, *Methylobacterium*, *Diaphorobacter*, *Comamonas*, *Mobilicoccus*, and *Fervidicoccus* were unique to and widespread among the pulmonary tuberculosis patients.

#### The phylum level composition of respiratory microbiomes

A total of 24 phyla were detected in the pulmonary tuberculosis samples, while 17 phyla were detected in healthy participants. Actinobacteria, Bacteroidetes, Proteobacteria, and Crenarchaeota were widely and abundantly distributed among nearly all of the samples. Firmicutes (37.02%), Bacteroidetes (29.01%), Proteobacteria (16.37%), Crenarchaeota (3.16%), and Actinobacteria (2.89%) were common in the healthy participants, while Firmicutes (41.62%), Bacteroidetes (7.64%), Proteobacteria (17.99%), Actinobacteria (21.20%), and Crenarchaeota (7.5%) were common in the pulmonary tuberculosis patients. Chlamydiae, Chloroflexi, Cyanobacteria/Chloroplast, Deinococcus-Thermus, Elusimicrobia, Euryarchaeota, SR1, Spirochaetes, Synergistetes, and Tenericutes were found in both the healthy participants and pulmonary tuberculosis patients, although they were rare in some samples. Aquificae, Caldiserica, Gemmatimonadetes, Lentisphaerae, Planctomycetes, Thermodesulfobacteria, and Verrucomicrobia were unique to the pulmonary tuberculosis samples. Moreover, in healthy participants, Deinococcus-Thermus, Bacteroidetes, and Fusobacteria accounted for 0.01%, 29.01% and 8.06%, respectively. However, in pulmonary tuberculosis patients, Deinococcus-Thermus increased to 0.93%, Bacteroidetes, and Fusobacteria decreased to 7.64% and 1.35%, respectively.

#### Several genera were uniquel to the respiratory tracts of pulmonary tuberculosis patients

Many genera were unique to in the sputum of pulmonary tuberculosis patients. As shown in Figure 3 and Table 1, *Phenylobacterium*, *Stenotrophomonas*, *Cupriavidus*, and *Pseudomonas* were found in nearly half of the tuberculosis patients we enrolled; furthermore, their total copies accounted for more than 1% of the total sequences from the sputum of pulmonary tuberculosis patients. Other genera such as *Sphingomonas*, *Mobilicoccus*, *Brevundimonas*, *Brevibacillus*, and *Diaphorobacter* were much more widely detected in pulmonary tuberculosis patients, even though they accounted for only a small number of sequences. Several rare genera were present in the sputum of pulmonary tuberculosis patients, such as *Thermus*, *Pelomonas*, *Methylobacterium*, *Comamonas*, *Lactobacillus*, *Thermobacillus*, *Auritidibacter*, *Lapillicoccus*, and *Devriesea.*

**Table 1:** The distribution of some genera that were uniquely found in the sputum of pulmonary tuberculosis patients

**Genera**	**α**	**β**
*Phenylobacterium*	13/31	2.15%
*Stenotrophomonas*	12/31	2.15%
*Cupriavidus*	16/31	1.60%
*Caulobacter*	5/31	1.56%
*Pseudomonas*	15/31	1.27%
*Thermus*	14/31	0.71%
*Sphingomonas*	16/31	0.66%
*Brevundimonas*	17/31	0.49%
*Pelomonas*	15/31	0.47%
*Acidovorax*	13/31	0.47%
*Brevibacillus*	16/31	0.36%
*Methylobacterium*	13/31	0.34%
*Diaphorobacter*	17/31	0.31%
*Comamonas*	14/31	0.26%
*Mobilicoccus*	20/31	0.24%
*Fervidicoccus*	13/31	0.21%
*Serpens*	5/31	0.19%
*Lactobacillus*	12/31	0.18%
*Thermobacillus*	12/31	0.16%
*Auritidibacter*	13/31	0.14%
*Deinococcus*	9/31	0.13%
*Lapillicoccus*	13/31	0.11%
*Devriesea*	13/31	0.11%

“α”: the number of pulmonary tuberculosis patients in whom sequences from the corresponding genera were found.

“β”: the percentage of sequences of the corresponding genera of all sequences found in pulmonary tuberculosis patients.

## Discussion

This study provides the first report on the microbial composition of the lower respiratory tract of pulmonary tuberculosis patients through the amplification of 16S rRNA V3 hyper-variable regions using bar-coded primers and pyro-sequencing by Roche 454 FLX. The results revealed that the microbial composition of the lower respiratory tract in pulmonary tuberculosis patients was more diverse (*p*<0.05) than in healthy participants. Charlson *et al* reported that the microbial composition of saliva or pharynx secretions can reflect the microbial communities in the lower respiratory tract, and their results showed that there is a topographical continuity of bacterial populations in the healthy human respiratory tract [17]. Therefore, we chose to use sputum and respiratory secretions in this study. However, the best samples to use would be lung lavage fluid, which perfectly reflects the lower microbial composition of the respiratory tract. However, obtaining lung lavage fluid is challenging, especially from healthy volunteers, because lung lavage is painful and may even be harmful. This may raise some ethical issues. In contrast, sputum and respiratory secretions are easily obtained through non-invasive, patients-friendly collection methods. Therefore, we chose to analyse sputum and respiratory tract secretions in our study. A previous study showed that fewer than 1% of commensal organisms are able to grow under laboratory conditions [18]; therefore, traditional cultivation-based strategies for analysing the complexity and genetic diversity of microbial communities are strongly biased. However, modern methods, based on barcoded primers and 454 pyro-sequencing allow for a thorough profiling of the microbiota of each enrolled person [19,20]. Published studies have also proved that the 16S rRNA V3 region sequence ideally suited for distinguishing all bacterial species to the genus level, except for closely related *Enterobacteriaceae*[21].

The lower respiratory tract microbiome of pulmonary tuberculosis patients was distinct from that of the healthy participants. As shown in Figures 1 and 2, the pulmonary tuberculosis patients formed a clear cluster that was separate from the healthy participants based on their microbiota. The phyla Bacteroidetes and Fusobactera were significantly underrpresented in pulmonary tuberculosis patients compared with healthy participants, while Actinobacteria was significantly overrepresented in pulmonary tuberculosis patients. Moreover, bacteria from the phylum Deinococcus-Thermus were widely distributed in pulmonary tuberculosis patients (15/31), but rarely found in healthy participants, and the phyla Aquificae, Caldiserica, Gemmatimonadetes, Lentisphaerae, Planctomycetes, Thermodesulfobacteria and Verrucomicrobia were unique to pulmonary tuberculosis patients. Figure 1 shows the genera *Klebsiella, Pseudomonas* and *Acinetobacter* were more common in pulmonary tuberculosis patients, and we postulated that these bacteria may aggravate the syndrome of pulmonary tuberculosis in these patients. Table 1 shows that the genera *Phenylobacterium, Stenotrophomonas, Cupriavidus, Caulobacter, Pseudomonas, Thermus* and *Sphingomonas* were unique to and widely distributed in patients with pulmonary tuberculosis.

The respiratory tract microbiota of pulmonary tuberculosis patients, who suffer from chronic infection, might be important in the pathogenicity of this disease. The variety of bacterial genera especially the presence of some abnormal genera in the sputum of pulmonary tuberculosis patients suggested that the pulmonary tuberculosis patient lung is an ecological niche that can support the growth of a high variety of bacteria, especially certain abnormal bacteria. These abnormal genera reportedly widespread in the environment, and some of them have even been reported to be associated with some infectious diseases [22-27]. Coenye *et al* also reported the isolation of unusual bacteria from the respiratory secretions of cystic fibrosis patients [22]. However, there are few reports on whether these organisms can cause human disease. The lower respiratory tract is an open system and can communicate freely with the environment. We speculated that, in pulmonary tuberculosis patients, the lung micro-environment may become more susceptible to colonisation by some foreign microbes. The host response to pathogens is characterised by rapid recognition combined with strong innate (i.e., inflammatory) and adaptive immune responses, enabling microbial eradication often at the cost of significant tissue damage. Furthermore, the host is constantly facing the challenge of discriminating between symbiotic and pathogenic bacteria to organise an appropriately an adaptive response [28]. These responses lead to the extensive fibrosis associated with recurring infections, possibly leading to a decreased clearance of lymph and lymph-associated particles from the infected region [29]. The lungs of individual patients typically contain diverse lesions with varied overall structures that change over time [3]. Ultimately, a strong host response to the clearance of *M. tuberculosis* may produce local lesions in the lung. This may in turn increase the possibility that foreign bacteria will colonise or grow in the lower respiratory tract. During the initial disease-causing invasion of the lung by *M. tuberculosis*, a strong host immune response may kill or clear some normal bacteria in the lower respiratory tract of pulmonary tuberculosis patients. This may be why the populations of many normal bacteria are decreased or absent from the microbiota of the pulmonary tuberculosis patients. At the same time, a strong host strong immune response against the pathogen may damage or produce lesions in the lung tissue, and consequently the micro-environment of the lower respiratory tract may favour colonisation or even host invasion by foreign microorganisms. These foreign bacteria may cooperate with *M. tuberculosis* to cuase additional damage to the lung tissue. In this model, although *M. tuberculosis* plays a central role in the disease, the other bacteria may assist in the destruction of the lung tissue, especially in active tuberculosis. If *M. tuberculosis* eliminated promptly, however, lung funtion can be restored. Further investigation will be required to determine whether pulmonary tuberculosis is the cause of increased foreign bacterial colonisation of the lower respiratory tract or vice versa (i.e., the presence of foreign bacteria aggravates the symptoms of pulmonary tuberculosis); is also possible that both occur simultaneously.

## Conclusions

This study demonstrated that the microbial composition of the respiratory tract of pulmonary tuberculosis patients was more complicated than that of healthy volunteers, and many foreign bacteria were found in the sputum of pulmonary tuberculosis patients. These foreign bacteria may participate in the onset or development of pulmonary tuberculosis.

## Methods

All of the procedures for the collection and handling of patient samples and data were reviewed and approved by the ethics committee of the Shanghai Pulmonary Hospital and Shanghai Jiaotong University School of Medicine, incompliance with the Helsinki Declaration of the World Medical Association. All study subjects provided written informed consent to participate in the study.

### Specimens

A total of 31 pulmonary tuberculosis patients, ranging in age 23 to 67 years old, with a age median of 39 years and a male/female ratio of 19/12, were recruited from the Shanghai Pneumonia Hospital. All patients were free of HIV. The patients were clinically diagnosed with pulmonary tuberculosis based on sputum smear, sputum culture, and computed tomography results. The sputum samples were collected after the patients had been admitted to the hospital. A portion of the sputum sample was used for medical tests, and the remaining sputum was preserved for DNA extraction after the patients were confirmed to have pulmonary tuberculosis. Anti-TB drug treatment (isoniazid, rifampicin, ethambutol, pyrazinamide) and its efficiency as demonstrated by computed tomography, were also considered to confirm the diagnosis of pulmonary tuberculosis. None of the patients had taken antibiotics for at least 3 months before sampling. Of the 31 patients tested, 12 were sputum culture positive, 9 were sputum smear positive, 20 were clinically diagnosed with bilateral tuberculosis, 7 were clinically diagnosed with right pulmonary tuberculosis, 2 were clinically diagnosed with left lung tuberculosis, 1 was clinically diagnosed with tuberculosis pleurisy, and 1 was clinically diagnosed with tuberculosis bronchiectasis.

The healthy volunteers were recruited from the same region as the tuberculosis patients. A total of 24 healthy participants, ranging from 38 to 66 years old, with a median age of 55, and a male and female ratio of 13/11, were recruited from Shanghai, China. The volunteers had similar lifestyle and eating habits, nutritional status and physical condition, were free of basic pulmonary diseases, severe lung disease, severe oral disease, systemic disease and other known diseases such as obesity or diabetes, that could affect the microbial composition of the respiratory tract. Volunteers with a history of smoking or drinking were also excluded. The healthy participants had not taken any antibiotics for at least 3 months before sampling. The samples from healthy participants were a mixture of saliva and pharyngeal secretions collected by deep coughing in the early morning before gargling. By coughing, the community that was originally in the sputum was contaminated by the normal flora of the oral cavity and pharynx. (The detailed information of the pulmonary tuberculosis patients and the healthy participants were showed in Additional file 1).

### Establishment of a pyro-sequencing library and pyro-sequencing using the 454 platform

DNA extraction and PCR of the 16S rRNA V3 region were performed as described in our previously published article [20]. However, several additional modifications were made. Fresh sputum samples were chosen soon after routine tests confirmed the diagnosis of pulmonary tuberculosis. After liquefaction at room temperature for 1 hour in a sterilised sodium hydroxide solution, 3 ml of sample was aliquoted into three 1.5 ml Eppnedorf tubes, pasteurised at 83°C for 30 min, and further extracted using a Bacterial DNA kit (OMEGA, Bio-Tek, USA). PCR enrichment of the 16S rRNA V3 hyper-variable region was performed with the forward primer 5’-XXXXXXXX-TACGGGAGGCAGCAG-3’ and the reverse primer 5’-XXXXXXXX-ATTACCGCGGCTGCTGG-3’. The 5’ terminus of each primer contained a different 8-base- oligonucleotide tag (represented by “XXXXXXXX” in the primer sequence), while the sequence after the hyphen was used to amplify the sequences of the V3 end region.

To ensure that a sufficient quantity of PCR product was amplified, a two-step PCR strategy was used. The first step was carried out in a 25 μl reaction volume containing 2.5 μl of PCR buffer (TAKARA), 0.625 U ExTaq (TAKARA), 0.1 μl of BSA (TAKARA), and 2 μl of primer solution with 100 μmol of each forward and reverse primer and 50 ng of extracted DNA as a template; ddH2O was added to reach the final volume of the reaction. Touchdown PCR was performed as follows: 5 min at 94°C for initial denaturation, followed by 20 cycles of 1 min at 94°C for denaturation, 1 min at 65°C for annealing and 1 minute at 72°C for extension, with the annealing temperature decreasing by 0.5°C for each cycle. The reaction volume in the second step of the PCR was 50 μl and contained 5 μl of the product from step one as a template. The reaction also included 5 μl of PCR buffer (TAKARA), 1.25 U ExTaq (TAKARA), 0.2 μl of BSA (TAKARA), 24 μl of water and 200 μmol of each barcoded forward and reverse primer. The amplification was carried out for five cycles of 1 minute at 94°C for denaturation, 1 minute at 55°C for annealing, and 1 minute at 72°C, with the temperature maintained at 20°C after the reaction was complete. Sequencing was performed at the Chinese National Human Genome Centre in Shanghai using a Roche 454 FLX instrument. The resulting sequences were published as SRA accession SRA051957.

### Phylogenetic and statistical analysis

The datasets were taxonomically grouped using the RDP classifier (the naive Bayesian classifier of the Ribosomal Database Project) at a confidence level of 90% [30]. The gross sequencing data were first searched for the linker, primers, and their reverse complements using the platform provided by the centre. The identified primer sequences were trimmed from each sequence read. Sequence reads that did not contain the 5’-end primer were removed from the dataset. The same program was also used for barcode identification. Barcodes were identified within the first 25 bases of the reads. Sequence reads were binned into FASTA files based on their barcodes.

Individual sequences were aligned using the Aligner tool, and aligned sequences files for each sample were processed by complete linkage clustering using distance criteria. We used Uclust to cluster all of the sequences, with a cut-off value of 97%. After clustering, we used a representative sequence of each type as the OUT (operational taxonomic units), and the record of each OUT sequence included the number of sequences and the associated classification information. These data were used to calculate the Shannon diversity and evenness indices. Fast UniFrac was used to analyse the phylogenetic microbial communities of the two types of samples [31]. Statistical analyses were carried out in SPSS 19.0, heatmaps were drawn in R, and Shannon diversity indices were estimated using Estimate S Win 8.20.

## Competing interest

All authors: We do not have any commercial interest in this work and have no conflict of interest with respect to the work represented in this article.

## Authors’ contributions

ZC analysed the data and wrote and edited the paper; ZC, YZ, and YZ were involved in generating the data; SZ, HL and ST assembled the clinical data and performed sampling; and XG and ST were responsible for the overall concept, design, and conduct of the study. All authors read and approved the final manuscript.

## Supplementary Material

Additional file 1 The detailed information of the pulmonary tuberculosis patients and the healthy participants. Click here for file
